# A Survey on Distributed Fibre Optic Sensor Data Modelling Techniques and Machine Learning Algorithms for Multiphase Fluid Flow Estimation

**DOI:** 10.3390/s21082801

**Published:** 2021-04-15

**Authors:** Hasan Asy’ari Arief, Tomasz Wiktorski, Peter James Thomas

**Affiliations:** 1 NORCE Norwegian Research Centre AS, 5008 Bergen, Norway; peth@norceresearch.no; 2 Department of Electrical Engineering and Computer Science, University of Stavanger, 4036 Stavanger, Norway; tomasz.wiktorski@uis.no

**Keywords:** multiphase fluid flow, machine learning, speed of sound, distributed acoustic sensor, distributed temperature sensor

## Abstract

Real-time monitoring of multiphase fluid flows with distributed fibre optic sensing has the potential to play a major role in industrial flow measurement applications. One such application is the optimization of hydrocarbon production to maximize short-term income, and prolong the operational lifetime of production wells and the reservoir. While the measurement technology itself is well understood and developed, a key remaining challenge is the establishment of robust data analysis tools that are capable of providing real-time conversion of enormous data quantities into actionable process indicators. This paper provides a comprehensive technical review of the data analysis techniques for distributed fibre optic technologies, with a particular focus on characterizing fluid flow in pipes. The review encompasses classical methods, such as the speed of sound estimation and Joule-Thomson coefficient, as well as their data-driven machine learning counterparts, such as Convolutional Neural Network (CNN), Support Vector Machine (SVM), and Ensemble Kalman Filter (EnKF) algorithms. The study aims to help end-users establish reliable, robust, and accurate solutions that can be deployed in a timely and effective way, and pave the wave for future developments in the field.

## 1. Introduction

Increasing field recovery is essential in the oil and gas industry. Equinor, a Norwegian state-owned energy company, estimates an untapped potential of around four billion barrels of oil from a 10% increase of oil recovery on the Norwegian Continental Shelf (NCS) alone [1]. Assuming the average oil price in the first quarter of 2021 around 61 USD per barrel [2], this equates to an economical value of 24.4 billion USD per percent of increased recovery; an enormous revenue increase from one region alone. In addition, there are also environmental benefits for such recovery enhancements, including: reducing carbon footprints due to production, and reducing the need for new oil field developments [3].

Production optimization is defined as the maximization of short and long-term production of oil, while at the same time minimizing production costs [4]. A typical starting point for production optimization is through continuous monitoring of the downhole production well variables (e.g., Water in Liquid Ratio (WLR), Gas Volume Fraction (GVF), fluid flow rate, water or gas breakthrough, and sand production). Typically, these measurements are then combined with simulations in order to optimize production control parameters (e.g., Inflow Control Valve (ICV) and/or Inflow Control Device (ICD) parameters, pressure setting, and controlling water/gas injection) for stimulating production [5]. It is a rigorous process with a continuous loop of monitoring, simulating, and optimizing the production of oil, while preventing and addressing anomalies and production disturbances. Therefore, it is crucial to have robust, reliable, and accurate monitoring capabilities to achieve the most optimized oil production system.

Some of the most powerful approaches for optimizing oil production employ multiphase fluid flow monitoring [6,7,8,9]. Such techniques are used to estimate and monitor the phase-fraction of oil, water, and gas inside the production wells. Metering devices, such as optical flow meter [10], Electrical Impedance Tomography (EIT) [11], differential pressure meters [12], and gamma densitometer [13] are used to estimate the multiphase fluid flow. It should be noted that some of the flow meter devices [14,15] use radioactive sources, thus, they require careful supervision during installation, operation, and disposal, not to mention a permit and experts for handling the radioactive materials [7]. Other non-radioactive devices such as the optical flow meters, however, are expensive and only provide a point-based measurement. For long-range pipeline and well applications, this significantly limits the monitoring capabilities of the overall system.

An inexpensive, non-invasive meter solution, called the Virtual Flow Meter (VFM), can also be used for multiphase flow estimation. VFMs combine pressure, temperature, and other process control data from an existing sensors with multiphase fluid models, in order to estimate the fluid flow rates. However, the VFMs require extensive parameter tuning and active sensor calibration [16,17,18]. Parameter tuning in VFMs becomes complicated due to the complexity and ever-changing variables in the downhole environment. As a result, VFMs have not experienced widespread adoption as a multiphase flow meter solution [7].

**Figure 1:**
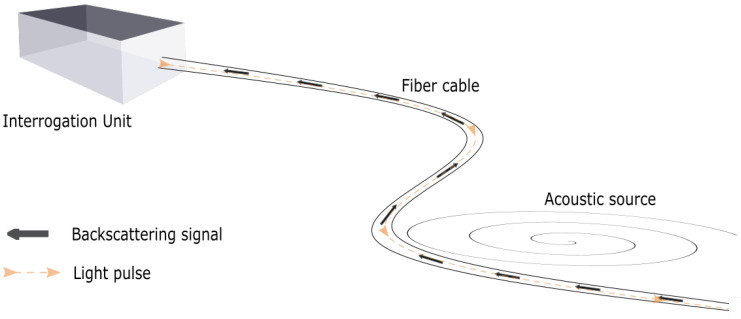
The Distributed Acoustic Sensor carries light pulse travelling inside the fibre cable that are backscattered to the Interrogation Unit that recovers the acoustic signal profile along the cable.

Distributed fibre optic sensors are receiving an increasing amount of attention. The sensors consist of a passive optical fibre and an interrogator unit located at one end of the fibre, depicted in Figure 1. Such systems are able to measure parameters at every position where the fibre optic cable is installed. They have been developed to measure temperature [19], strain [20], acoustic [21], and even chemical [22] parameters, such as humidity [23] and the presence of oil [24] in a distributed manner. The sensors operate by using the interrogator to send pulses of laser light along with the fibre. As the light pulses propagate, they interact with the glass fibre in several different ways, leading to the generation of three distinct types of scattered light (propagating back to the interrogator), known as Rayleigh, Raman and Brillouin scatter. Each backscatter type can be distinguished by its frequency content and is influenced by different physical parameters, forming the basis of different Distributed Sensors (DSs), such as Distributed Temperature Sensor (DTS) [19] and Distributed Acoustic Sensor (DAS) [21].

In 1996, DTS technology began to find its use in real-world applications [25]. Subsequently in 2009, a DAS system was used to successfully measure an increase in oil production following a fracking intervention [26]. Since then, the DSs have formed the basis of a great deal of research, both in the development and understanding of the technologies [27], as well as their practical application. Due to their long-range and distributed monitoring capabilities, the distributed fibre sensors have been deployed for Vertical Seismic Profiling (VSP) [28], hydraulic fracture detection [29], early-warning systems for earthquake and seismic activity monitoring [30], traffic pattern analysis and monitoring [31], and in-well flow profiling that not only used in the laboratory or field trials but also in real-time operations [6,32,33,34].

In general, applied DS systems require advanced data processing algorithms to estimate physical quantities of certain parameters within their surroundings. For the multiphase fluid flow measurement, these algorithms are often needed to be carried out in real time. This challenge is particularly great for the DAS systems where the raw data quantities can often exceed 1 GB/s. Speed of sound estimation from acoustic data [9] and Joule-Thomson coefficient from temperature data [35] are the state-of-the-art techniques that are used to estimate the multiphase fluid flow from DS data. In recent years, the ability to perform similar tasks using data-driven machine learning algorithms have been explored. For example, Convolutional Neural Network (CNN) and Artificial Neural Network (ANN) have been used to perform flow regime classification and multiphase estimation [36,37]. Subsequently, the ANN with Long Short-Term Memory (LSTM) algorithm has also been used to perform fluid flow rate estimation [38,39,40,41].

In this paper, we provide a structured and comprehensive review of the recent advances in the multiphase fluid flow estimation based on the distributed fibre optic sensor technologies. In Section 2 we begin by reviewing the challenges and importance of multiphase fluid flow and discuss existing technologies and devices that perform such measurement. In Section 3, we review the distributed sensor technologies and their working mechanism. In Section 4 we present the state-of-the-art in regards to data modelling algorithms that characterize multiphase fluid flows by combining DS measurements with physical flow modelling. Section 5 presents a similar state-of-the-art overview for machine learning-based algorithm for characterizing multiphase fluid flows. Finally, Section 6 discusses the performance and limitations of the state-of-the-art, in addition to potential future research directions.

To the best of our knowledge, this is the first paper that presents and analyzes the state-of-the-art in multiphase fluid flow characterisation with an emphasis on distributed fibre optic sensor and machine learning algorithms. There are several excellent reviews for multiphase flow estimation using other technologies, such as VFMs [17,18,42] and hardware-based flow meters [7,43].

## 2. Multiphase Fluid Flow

Multiphase fluid flows are defined as the simultaneous flow of more than one fluid phase through channels, such as porous media or pipelines [44]. Multiphase fluid flows in the context of the oil and gas industry, are the combinations of water, oil, and gas. Knowledge of the state of the multiphase flow can be used to optimize the production wells by, for example, changing the ICV or ICD settings in a way that maintains oil uplift and avoids problems such as water or gas coning, sand production, sharp pressure, or flow rate drops [9]. Moreover, with real-time knowledge over extended lengths of the flow column, anomalies can be detected as soon as they arise irrespective of their location, allowing for a targeted solution that can be implemented instantly without shutting down the whole production system. In this way, real time information about the state of multiphase flows provides a transformative capability for the reservoir and production engineers for reducing operational disturbances and maximizing oil production [9,10].

However, providing an accurate, reliable, and cost-effective measurement of multiphase fluid flow is a huge challenge in the oil and gas industry. In addition to the flow variables (such as flow pattern, phase density, fraction, and velocity), other factors are influencing the multiphase measurements, for example, (1) operating pressure and temperature, (2) pipe properties such as diameter, shape, inclination, surface roughness, and (3) the presence of other components such as valves, T-junctions, and bends [7,9,10]. The challenge becomes greater for the downhole in-well measurement, due to the High Pressure and High Temperature (HPHT) environments. Other complicating variables include the formation of emulsion and hydrate phases, as well as the presence of sand that flow carries from the formation.

The multiphase flow in a pipeline is characterised by the flow regime and the corresponding flow rate. The multiphase flow can be represented as the fraction of fluid phases flowing simultaneously at a given time and location. The flow rate, on the other hand, represents the volume of fluid flowing per unit time at a given location. They can be estimated using the speed of sound propagating in the fluid, flow velocity, pressure and temperature in a given flow regime, and fluid density.

**Figure 2:**
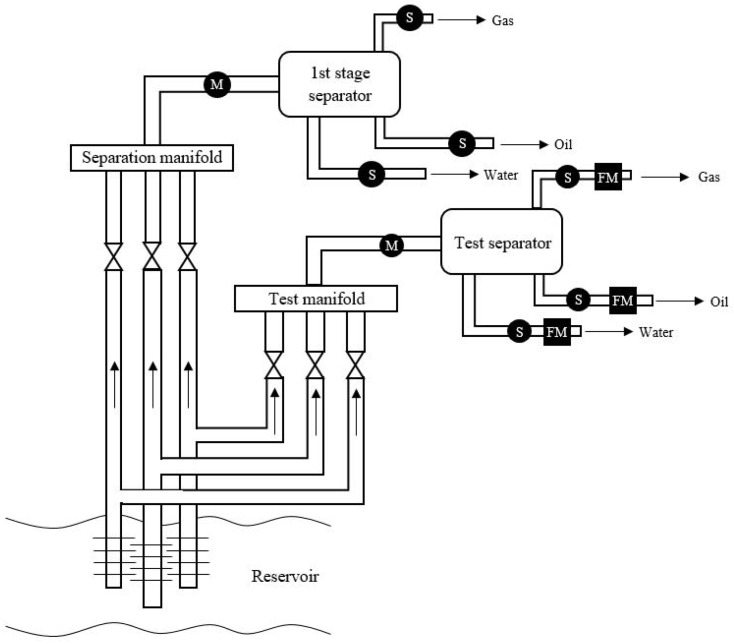
Schematic of a typical test separator within an oil production system. The multiphase flow is denoted as M while the single phase flow is denoted as S. Reprinted from ref. [7].

Several different approaches can be used to measure the multiphase flow, including but not limited to, conventional dedicated hardware-based flow meters [7,10,14,15,43], virtual flow meters [17,18,39,42,45,46,47], and distributed sensor flow estimations [8,37,40,48]. This paper focuses on providing a comprehensive review of the last approach, using distributed sensors with physical flow modelling and machine learning algorithms for multiphase flow estimation. A brief summary of the other two approaches is provided in the following subsections. For completeness, it must be stated that phase separation (followed by single-phase fluid flow measurement, depicted in Figure 2) can also be employed for characterising the multiphase flow [49]. While this type of multiphase fluid characterisation is accurate, it is extremely resource and time demanding. Therefore there has been a lot of R&D effort towards developing multiphase flow meters.

### 2.1. Hardware-Based Flow Meter

Conventional hardware-based multiphase flow meters (MPFMs), can provide accurate measurements of density, velocity, and phase fraction of fluid flow in the pipes. Electrical capacitance tomography [50], electrical impedance tomography [51], and optical tomography [52], are commonly encountered technologies that are used to build MPFM sensors. These technologies acquire a series of simultaneous images from a cross-section of the pipes, and cross-correlate these to determine the values of flow velocity [7].

Similarly, gamma densitometry is another technological advancement that can be used to build tomographic MPFM sensors. Such devices rely on the different ray attenuation properties of the different phases for performing the multiphase measurement [7]. While the gamma-ray instruments can provide very accurate measurements [53], their reliance on radioactive sources presents additional challenges with respect to installation, operation, and disposal.

Optical flow meters represent another family of MPFM sensors. They use the flow velocity and speed of sound of the flowing fluid to estimate the phase-fraction of the fluids. The flow velocity is obtained by tracking the vertical structures in the flow, and the speed of sound is obtained from the acoustic waves of the turbulent flow [10]. The optical flow meters are commercially mature devices and can provide reliable point-based measurements [9].

### 2.2. Virtual Flow Meter (VFM)

VFMs work by combining numerical models with available field data (e.g., pressure and temperature from both the bottomhole and wellhead of well chokes, as mentioned in [42]) to estimate the multiphase flow. Based on its modelling paradigms, the VFMs can be divided into first principles VFM and data-driven VFM. Flow modelling in near-well region, pipelines, and production chokes, together with pressure and temperature measurements are utilized by the first principles VFMs to estimate fluid flow rates [54]. The steady-state optimization algorithms, such as choke model, can be used to provide a point-based fluid flow estimation [42]. On the other hand, the dynamic state optimization algorithms, such as Kalman Filter, can be used to provide dynamic modelling of the first principle VFMs [55].

Data-driven VFMs, however, work by using machine learning algorithms to estimate the multiphase flow. The general framework is to fit the field data and mathematical models (lead by machine learning algorithms),without basing them on exact understanding of the physical parameters and information of the production wells and the reservoir. Several machine learning algorithms, including Support Vector Machine (SVM), LSTM, ANN, and gradient boosting algorithms [39,56,57,58,59], can be used to perform the multiphase estimation.

Combining both first principles and machine learning algorithms can help to improve accuracy as well as the transparency of the VFM-based approaches, providing insight into the physical origins of the results [60]. For an in-depth review of the first principles and data-driven VFMs, we direct the reader to an excellent review by Bikmukhametov et al. [42].

## 3. Distributed Sensor Technologies

Physically, a distributed fibre optic sensor is a passive cable that’s capable of spatially resolved measurements, similar to that achievable with a densely spaced array of point sensors [61]. As a surveillance and monitoring infrastructure, it provides a lot of benefits, for example, (1) it is less difficult to install compared to installing a large number of sensors, (2) it works in passive mode reducing the power source utilization and is easy to maintain since most of the system’s complex elements can be made easily accessible, and (3) it can work in harsh HPHT environment with sufficient coating materials.

### 3.1. Distributed Sensor Working Mechanism

**Figure 3:**
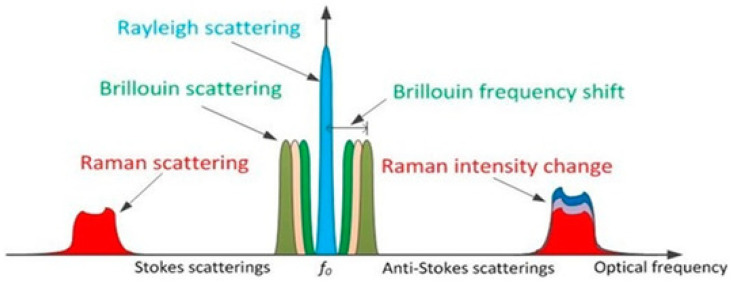
The backscattering phenomenons used for distributed fibre optic sensing. Reprinted from ref. [22].

The main component of Distributed Sensors (DSs), in addition to the fibre cable, is the Interrogation Unit (IU) or Interrogator. It sends pulses of laser light through a fibre optic cable and then analyses the properties of the elastic and inelastic backscattered light [62] as depicted in Figure 1. These measurements are characterised by the frequency, phase, and amplitude properties of the backscattered light [6], that are in turn related to temperature, pressure, vibration, and strain changes within the cable and therefore it’s surroundings. The distributed sensors work based on three distinct scattering processes that take place as laser pulses interact with the glass fibre: Rayleigh backscattering [63], Raman backscattering [64], and Brillouin backscattering [65], depicted in Figure 3.

Environmental conditions, such as temperature and strain changes, directly affect the backscattered signals. The DTS exploits these phenomenons by measuring the intensity of anti-Stokes Raman scattering signal, as a function of the local temperature changes within the fibre cable. The DAS, however, operates differently. The Rayleigh backscattering used by DAS is modulated in intensity and phase as a function of acoustic/strain perturbations [66]. Since the fibre strain is also influenced by the temperature, Rayleigh backscattering can also be used as a second form of DTS. Unlike Raman-based DTS, the Rayleigh DTS uses the same fibre type as that for DAS, meaning that a single fibre can be used to perform DTS and DAS together [67]. For a more detailed explanation of these backscattering phenomenons and fibre-based distributed sensors, we suggest excellent distributed sensor reviews by Gohari et al. [6], Lu et al. [22], and Schenato [66].

It is important to highlight that for measurements at any given location, the DAS data have a low Signal to Noise Ratio (SNR) relative to their array-point sensor counterparts. One of the strengths of DAS, over the point-based sensors, is their ability to provide data continuously along the cable. This feature, together with the ability to make measurements with high temporal frequency, indicates large data rates. This data rate is ultimately only limited by the time taken for the backscatter from each pulse to leave the fibre, before the next pulse can be introduced. This in turn highlights the importance of powerful and efficient data processing algorithms. One common method of reducing the influence of noise is through an algorithm known as F-K filtering.

The design of the DAS cable itself can have a significant effect on the system performance (as can the cable installation itself) that should be done to optimise the signal coupling. Helically wound fibre cable is an example of how cable design can influence the sensing properties [68]. Firstly, the helical trajectory through the cable ensures the cable has “broadside” sensitivity to disturbances perpendicular to the cable. It is more sensitive compared to the straight cables which mostly being influenced by axial disturbances. Secondly, the fibres are wound about a compliant material that serves as a sensitivity amplifier [68]. In addition, engineered-fibres are becoming the subject of much research in recent years, where the motivation is to create fibres with enhanced Rayleigh scattering, therefore increasing the signal [69]. Such engineered-fibres are particularly useful for increasing the range of the DAS systems, that are typically limited to a few 10 km by attenuation of the light by the glass. It is worth noting that the DSs are not meant to be the replacement of conventional point flow meter devices, but they work as complimentary equipment to provide distributed measurements in a cost-effective way.

### 3.2. Applications for Distributed Sensors

The early applications for DSs technologies were in DTS, which were used to measure temperature and pressure changes in a field trial in West Coalinga Field, CA, USA, in 1996 [25]. Since then, significant improvements have been made both in technologies and hardware systems. The DTSs have been used for transformer monitoring [70], wildfire behavior characterization [71], leakage detection [72], structure monitoring [73], fire detection [74], and cooling effect and temperature log in oil and gas industry [75] as was mentioned in [76].

**Figure 4:**
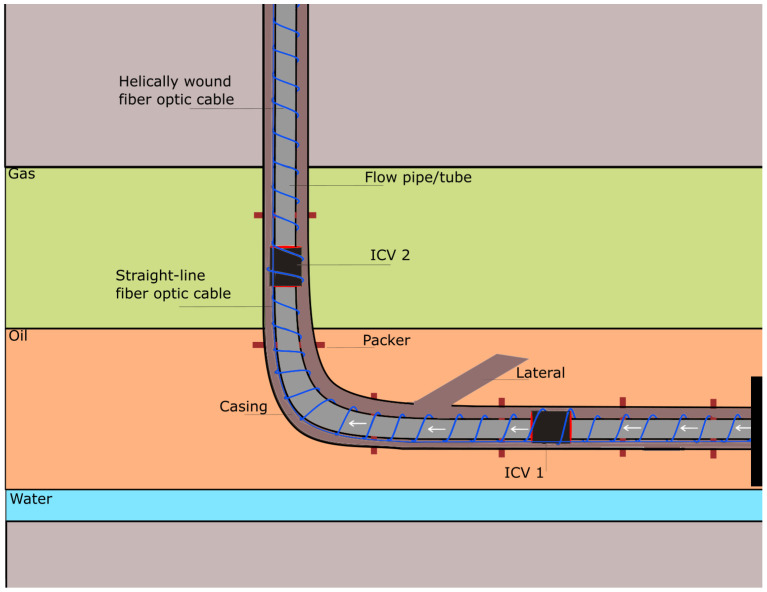
A sketch of a smart well with ICVs and fibre cables mounted around the flow pipe.

DAS systems, began to emerge in the late 2000s, have been used for early-warning system for earthquake and seismic activity monitoring [30], hydraulic fracture detection [29], traffic pattern analysis and monitoring [31], gas leak detection [77], pipeline surveillance [78], Vertical Seismic Profiling (VSP) [28], Steam Assisted Gravity Drainage (SADG) monitoring [79], and in-well flow profiling that not only used in the laboratory or field trials but also in the real time operations [6,8,32,33]. Combining DTS and DAS data has also been explored, for example, to address the three-phase flow estimation of oil, water, and gas for the downhole well simulations [8], which was less accurate and seems unsolvable when only using DAS or DTS alone. Figure 4 shows a sketch of smart wells with several ICVs and fibre-based distributed sensors as a straight-line and helically wound cable around the pipe.

A single DAS IU can generate 20 Terabyte (TB) of data per day [80]. In an experiment by Ajo-Franklin et al. [80], 128 TB data was generated by sampling 12,000 channels at a frequency of 500 Hz over a period of 3 months. It means that as a permanent monitoring device for an in-well downhole operation, a single fibre-based distributed sensor can generate more than several Petabytes (PB) of data during its lifetime. Therefore, advanced data management strategies, data compression algorithms, feature extraction techniques, including comprehensive signal processing algorithms are required to process the DS data to provide a realtime monitoring solution over a long period of time.

## 4. Physical Flow Modelling

Physical flow modelling can be defined as using physical phenomena to extract valuable information from a given data. In the case of multiphase fluid flow estimation, the physical flow modelling is used to approximate the changes of physical phenomena to the value and phase-fraction of the multiphase fluids. Temperature changes, flow velocity changes, and speed of sound changes, are some of the physical phenomena that fluctuate when multiphase (or even single-phase) fluids flow. Distributed fibre optics can be be used to measure these values, with consideration of the complexity of data processing required being dependent on the DS type and the parameter of interest. For example the processing workflow for measuring flow temperature with DTS [75] is relatively simple compared to measuring speed of sound and flow velocity using DAS data [8,9].

**Figure 5:**
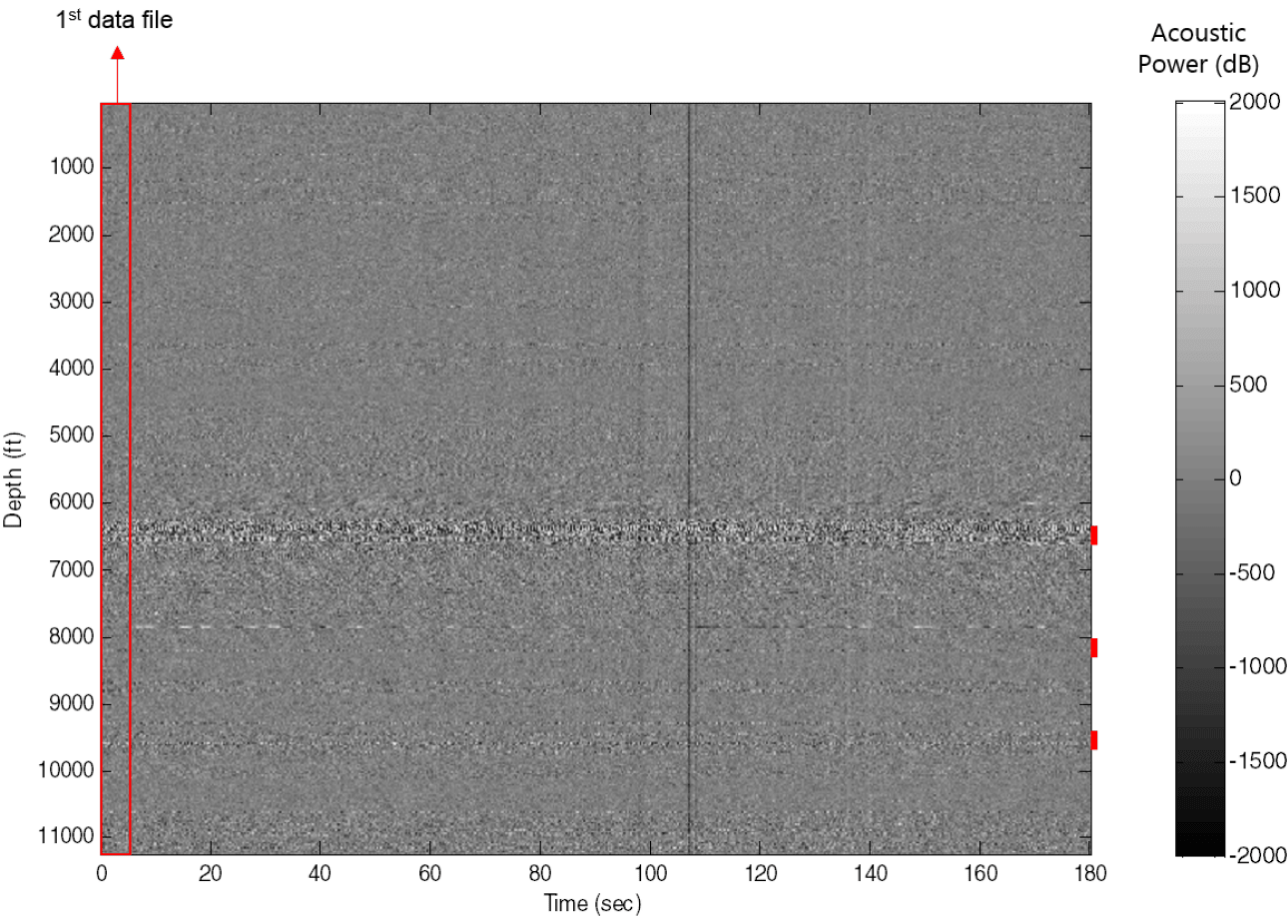
Raw acoustic data collected from a multilateral well. Reprinted from ref. [34].

The common framework of using physical flow modelling for estimating the multiphase flow from distributed sensor is as follows:

1. gather a block of data as measured intervals, corresponding to a specific range of time and location, see Figure 5,
2. extract the physical parameter values, including speed of sound and flow velocity (more can be found in Table 1), then
3. estimate the multiphase values using extracted physical flow features and data from fluid mixture databases. Examples of publicly available database can be found in [81,82,83,84].

### 4.1. Data Acquisition

The DS data must be processed partially due to the size. As was mentioned in [34], a one-minute of DAS data often could not be loaded to regular desktop PC hardware. Therefore, the data should be divided into series of blocks for processing; the size of the blocks will represent the spatial and temporal resolution of the flow profile. It should be pointed out that spatial overlapping of consecutive blocks is often employed during processing in order to increase the spatial resolution and enhance the repeatability [48].

**Figure 6:**
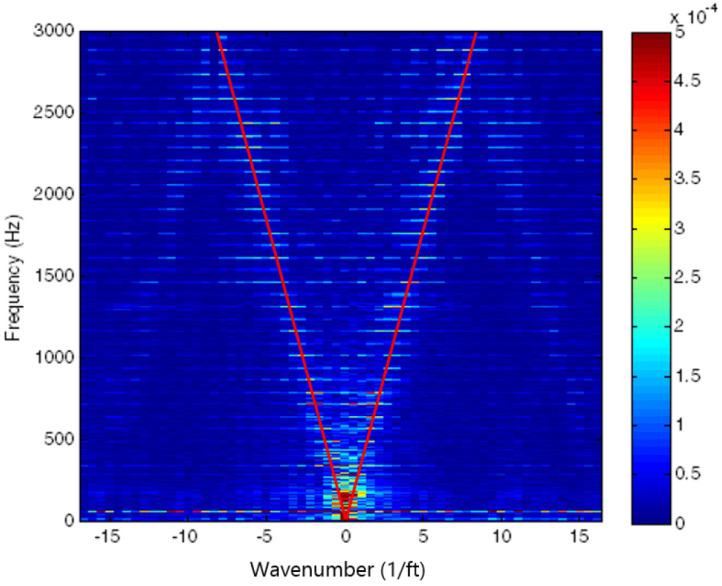
F-K plot generated using 2D-FFT with V-shape line fitting. Reprinted from ref [34].

After dividing the data into several blocks, it is often required to transform each blocks of DAS data from time-space domain, corresponding to the time of data being recorded and the spatial locations within the fibre cable, to the frequency and wavenumber domain, also called F-K domain. The two dimensional Fast Fourier Transforms (FFT) algorithm [40] can be used to perform such transformation (F(f,k)) as depicted in Figure 6. It is defined in Equation (1) where t,x denote the time and location of the input data, while f,k denote the frequency and wavenumber, respectively. Please note that the complex physical values, such as speed of sound and flow velocity can be extracted from the F-K domain.

(1) $$F\left(f,k\right)=\int_{}\int_{}f\left(t,x\right)e^{-i(kx-2\pi ft)}\;dx\;dt.$$

### 4.2. Physical Flow Data Extraction

#### 4.2.1. Speed of Sound

**Figure 7:**
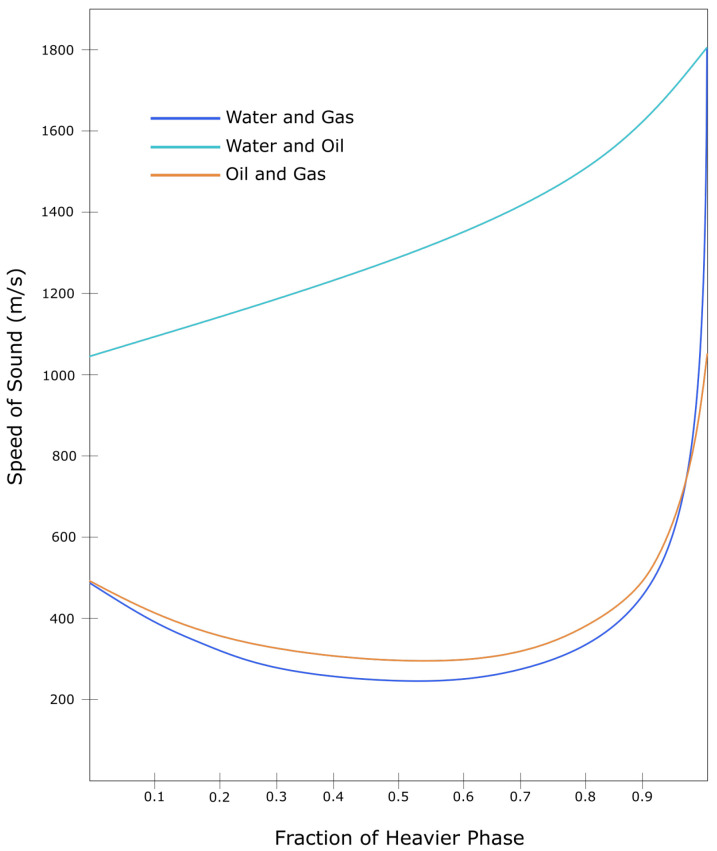
The relation between speed of sound and fraction of fluid mixture. Adapted from ref. [5].

Sounds travel at different speed depending on density, pressure, temperature, and molecular structures within the travelling medium. For multiphase fluids, sound travels faster within the water phases than in the oil and gas phase components [34]. The Speed of Sound (SoS) measures how fast sounds travel within a medium. In fact, the SoS has been used as a strong feature to estimate the type of medium where the sound travels. The method is called phase estimation [9,48]. Figure 7 shows a possible range of mixture fluid percentages given the SoS within those fluids.

**Figure 8:**
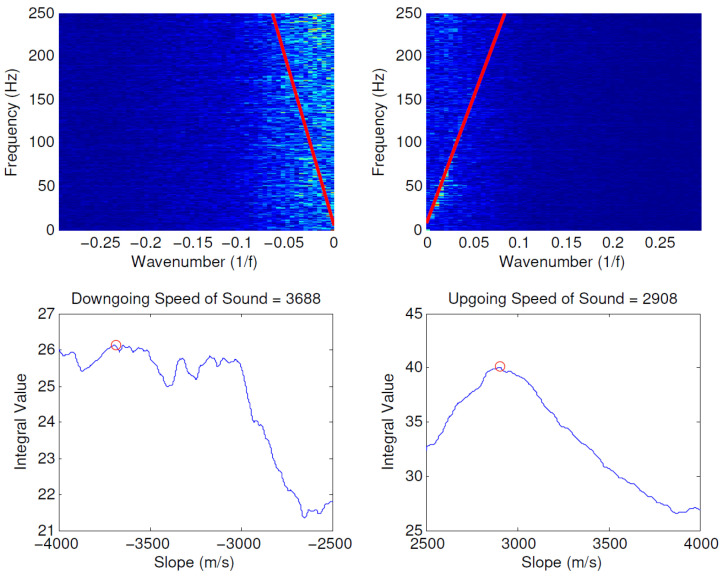
Comparison of the speed of sound between a clear line image (**left**) and not a clear one (**right**). Reprinted from ref. [34].

The SoS can be measured by applying a line fitting algorithm [40,92] from an F-K plot of the DAS data, depicted in Figure 8. It is based on the slopes of the lines in the F-K domain, where the frequency and the high Fourier coefficients form the speed of sound [34]. It is formulated in Equation (2) where $c_{m}$ denotes the multiphase SoS, while λ, *f*, and *k* denote wavelength, frequency, and wavenumber respectively. It will be recalled that $c_{m}$ from the slope lines are consisted of the upgoing ($c_{u}$) and downgoing ($c_{d}$) SoS from the propagating acoustic wave. The positive slope of the line represents the $c_{u}$, while the negative one represents the $c_{d}$.

(2) $$c_{m}=\lambda f=\frac{2\pi f}{k}.$$

#### 4.2.2. Flow Velocity

The flow velocity is defined as the speed of travelling fluids inside the pipe; the flow rate is calculated from the flow velocity by including the flow pipe diameter in the calculation. The flow velocity is derived from the Doppler Effect (or Doppler Shift) principle of $c_{u}$ and $c_{d}$ [5]. It is important to highlight that when a flow approaches a sensor (at a given location), the sound waves that reach the sensor have a shorter wavelength and a higher frequency. However, when the flow moves away from the sensor, the sound waves that reach the sensor have a longer wavelength and lower frequency. This phenomenon is called the Doppler Effect. It measures the changes in an apparent frequency of a wave when the flow (of the acoustic source) moves relative to a stationary sensor location. The flow velocity can be calculated using this phenomenon. It is defined in Equation (3) where *v* is the flow velocity and $c_{d}$ is assumed to be negative, see [5] for a more comprehensive derivation.

(3) $$v=\frac{c_{u}+c_{d}}{2}.$$

#### 4.2.3. Joule-Thomson Effect

**Figure 9:**
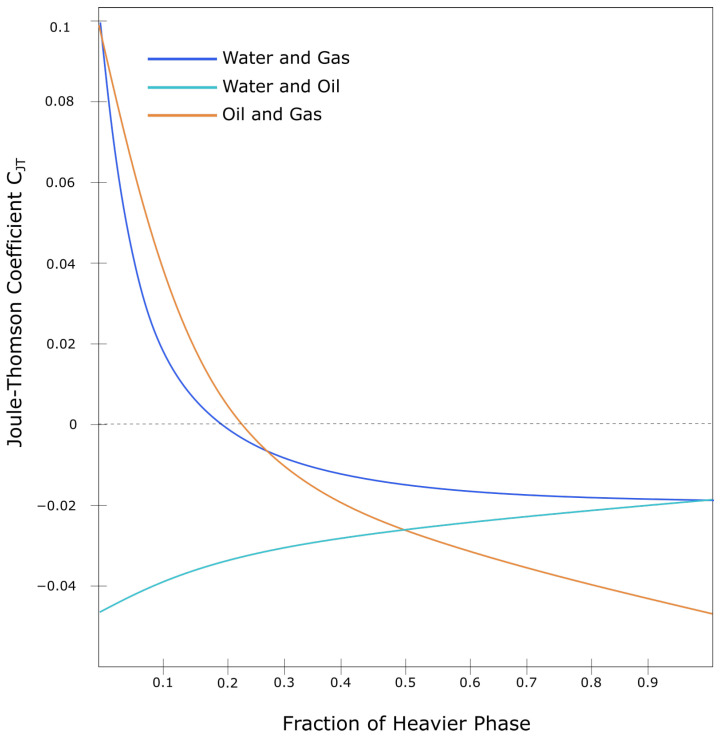
The relation between Joule-Thomson coefficient and fraction of fluid mixture. Adapted from ref. [34].

The Joule-Thomson effect is characterized by temperature changes that happens when fluids flow through a valve or porous plug with no heat exchange in the environment [34]. Wang’s work [93] has been focused on analyzing Joule-Thomson Coefficient ($C_{JT}$) using DTS data. The $C_{JT}$ in the fluid mixtures is defined as a function of well temperature and pressure, compressibility factor, and fluid mass-weighted phase fractions, more detail can be found in [34]. Figure 9 shows the $C_{JT}$ as a function of phase fraction and can be used to estimate accurate two-phase flow from water, oil, and gas.

### 4.3. Multiphase Estimation

Determination of the SoS, flow velocity, and Joule-Thomson coefficient represent the state-of-the-art with regards to fluid flow characterisation using DAS and DTS data. These data, are then calibrated using information from fluid mixture databases to calculate multiphase flow parameters such as WLR and GVF. The National Institute of Standards and Technology (NIST) in the US has provided a comprehensive database of thermophysical and geophysical properties of hydrocarbon mixtures [82,84].

**Figure 10:**
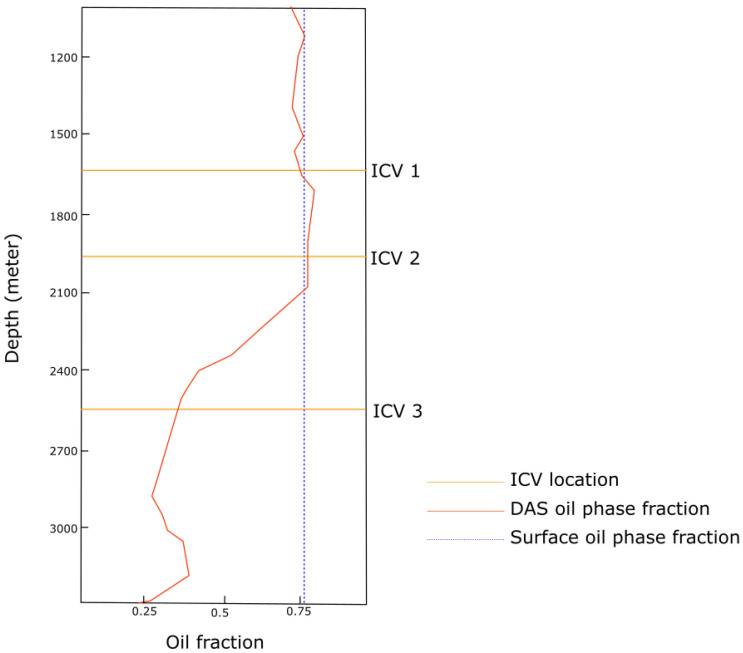
Example of two-phase flow characterisation using DAS data that has been used to calculate the oil fraction. The figure also shows the location of the ICVs.

The physical values, when accurately estimated, can provide high accuracy multiphase flow information as depicted in Figure 10. However, prediction uncertainties are expected due to factors such as volatilities of the surrounding physical environment, sensor noise, systematic errors in the measurement method, corruptions within the data, and other problems during value extraction process that might arise [94]. Thus, including error estimations and uncertainty values, when providing the multiphase information for realtime monitoring solution is often a requirement.

A summary of physical flow modelling techniques that have been used with distributed fibre optic data for flow rate and multiphase estimation is provided in Table 1. Johannessen et al. [85] provided an early work on using DAS data to extract qualitative information on the flow regime, speed of sound and an estimate for flow velocity in some part of the wells. Even though quantitative analysis in [85] was limited, the work showed an interesting qualitative analysis that tied together well acoustic signatures and well behaviour. Xiao et al. [48], also presented DAS data obtained using cables installed within producing wells. The work presented several data analysis technique for modelling and enhancing DAS performance, including RMS of acoustic energy, amplitude estimation, FFT transformation, and SoS with flow analysis. As the number of the spatial channel of the recorded DAS data increased, the SoS calculation can provide higher accuracy and precision, influencing the overall accuracy of estimating fluid flow rates. Finfer et al. [86] provided experimental results from single- and multiphase tests for assessing DAS suitability for monitoring fluid velocity and flow composition. In [86], F-K transformations were employed for measuring flow velocity from DAS data, The paper also proposed the use of multiphase multipoint flow sensing and provided practical guidance on how to set up a DAS system for real-time multiphase measurement. Fidaner et al. [87], on the other hand, developed a forward model to connect between two-phase flow in the wellbore and DAS data using a set of analytical expressions, such as physical fluid mechanism, propagation of the acoustic signal, and phase changes in optical signals due to pressure change. The wavelet analysis method was used to capture the most relevant components of DAS data for multiphase flow rate estimation. These components were then trained using ANN to obtain a more realistic flow rate estimation model.

The work of Abukhamsin et al. [8,34] covers many measurement aspects discussed in this review. In particular Abukhamsin et al discussed the use of DAS, DTS, and the combination of the two on addressing the challenges of characterizing three-phase flows. Even though, the DTS was derived from commercial thermal simulator [34], the work shows a promising result on combining SoS from DAS and $C_{JT}$ from simulated DTS to provide an accurate multiphase estimation. Hemink et al. [88], on the other hand, showed that $C_{JT}$ from actual DTS does not always provide reliable results as a straightforward thermal model to identify gas-injection. Instead, the work proposed an improvement by considering the temperature response measured by DTS where the fibre is clamped and bending away from the tubing. Using the DTS trace, the identification of the annular-fluid interfaces (brine/gas, gas/flowing gas) was possible, as well as the depths of active lifting points. Shirdel et al. [89] employed several signal processing algorithms, including DAS spectrogram, DTS and DAS waterfall analysis, and steady-state injection, to interpret the DTS and DAS data to provide a quantitative step-injection-flow profiling. It shows that those algorithms are tied together with an independent physical principle related to multiphase flow, acoustic effects, data array, and others. The work can be used to analyze complex flow regimes and heat transfer of wet-steam flow in horizontal wells providing a good basis for benchmarking multiphase estimation algorithms.

Another flow-loop experiment and simulation model was presented by Soroush et al. [90]. The work focused on analysing the potential of fibre optics technology to perform inferential multi-phase flow measurement. The results showed that the flow regime and existence of gas-phases could be determined by DAS-based on the signal frequency content. These results are crucial for SAGD wellbore monitoring on detecting steam breakthrough. Another work is from Cerrahoglu et al. [91] on identifying cluster flow from DAS and DTS on horizontal dry gas-producing wells from the HPHT environments. They showed that using SoS analysis from the cable bottom section, nearly 50% of the total gas rate comes from below the cable. A result that might be skewed when performing a full spatial channel analysis based on SoS calculation.

## 5. Machine Learning

**Figure 11:**
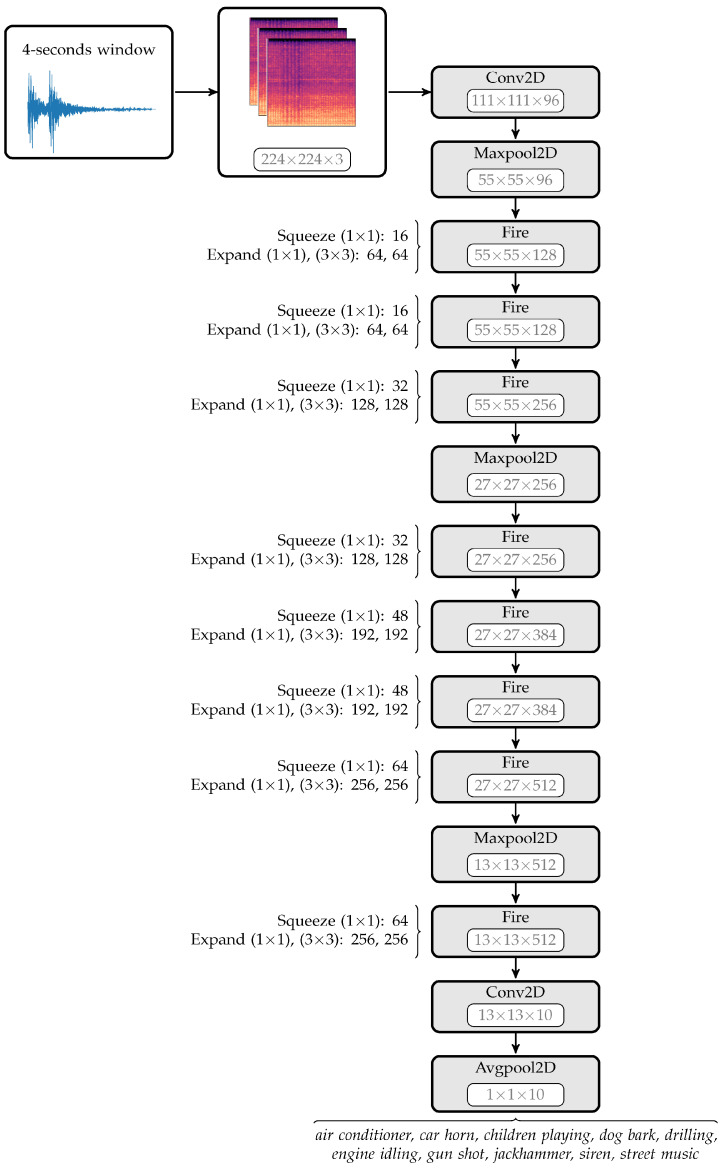
An example schematic for modelling DAS data using a preprocessed spectrogram plot on CNN-based models. Reprinted from ref. [97].

Advances in the machine learning field in the past few years have generated a lot of interest for potential applications within the oil and gas industry, especially in the realm of production monitoring and automatic surveillance. For example, several machine learning-based techniques have been used for multiphase flow and flow rate estimation, including feed-forward Neural Network (NN) [58], Recurrent Neural Network (RNN) [39,41], Support Vector Machine (SVM) [57], gradient boosting algorithm with regression trees [95], and Kalman Filter (KF) [41]. An example schematic of modelling the DAS data using CNN algorithm can be seen in Figure 11. Most of those works, however, used the machine learning algorithms on the VFM domain, while only a handful of research (including the work of Jalilian et al. [96], Silkina [36], and Vahabi et al. [37,40]) have been focused on using machine learning on the DS data for flow rate and phase estimation.

**Figure 12:**
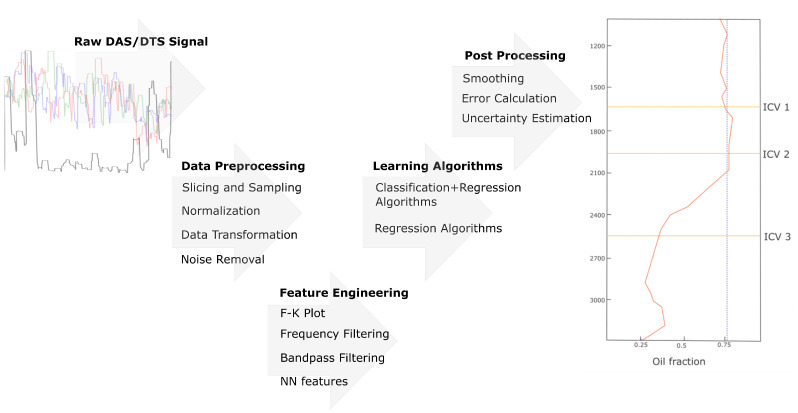
The common workflow of machine learning-based techniques for multiphase fluid flow characterisation.

Even though the actual implementations may vary, the common pipeline that is used for multiphase estimation based on the machine learning techniques can be simplified as has been depicted in Figure 12. It starts with the data acquisition and preprocessing, and concludes with an inference process which can include prediction, smoothing, and extrapolation.

### 5.1. Data Preprocessing

According to Forbes, more than 60% of a data scientist’s time is utilized on the data understanding and preprocessing step [98]. It is the main backbone within the overall machine learning workflow. The data preprocessing includes gathering, cleansing, slicing, and transforming the input data to be forwarded and processed into the next step [37,99]. With the large size of the DS data, offline preprocessing sometimes is required to simplify the learning process and speed up the overall implementation, similar to that demonstrated in Vahabi et al.’s work [37].

Several techniques can be used to speed up the data preprocessing step. For example, GPU-based implementation can be used for preprocessing the DAS data [100]. Generative Adversarial Network (GAN) can be used to simplify the data generation process which can help reduce the total processing time [101]. Moreover, reducing the data transformation procedures and only use simple bandpass filtering can also help speed up the data preprocessing step, similar to Shi et al.’s work [99].

### 5.2. Feature Engineering

In machine learning, features are defined as the measurement values that can be obtained from the object of interest. Acoustic amplitude/gain, temperature, time, and measurement locations, are some of the features that can directly be obtained from the DSs data. Depending on the objective of the machine learning task, the features can be used to classify an object or to predict the next possible changes within the particular object of interest.

Feature engineering, on the other hand, refers to the techniques used to transform the existing features to a new domain where the new features are generated. These new features can be used to enrich the ability of a machine learning model to achieve a better outcome. Based on the way new features are generated, they can be divided into handcrafted and non-handcrafted feature engineering techniques [102].

The handcrafted feature engineering techniques derive new properties using various algorithms or physical formulations based on the understanding of the physical phenomenons captured in the input data. The FFT transformation [37], bandpass filtering [99], F-K filtering [103], and physical flow feature transformations in Section 4.2 are considered handcrafted feature engineering techniques. On the other hand, the non-handcrafted techniques generate new properties without understanding the physical phenomenons surrounding the input data. These techniques perform cross-correlation, feature combination and multiplication, and high-dimensional transformation of the input data. The aim is to provide new representations of the input data that are useful for achieving accurate prediction. Multi Layer Perceptron (MLP) [104], Binary Descriptor [105], and multi-stage CNNs [37] are considered as the non-handcrafted engineering techniques that can be used for multiphase estimation. Dimensional reduction algorithm, such as Principle Component Analysis (PCA), can be used to reduce the number of features while selecting highly relevant features for multiphase estimation objective [57,106].

**Figure 13:**
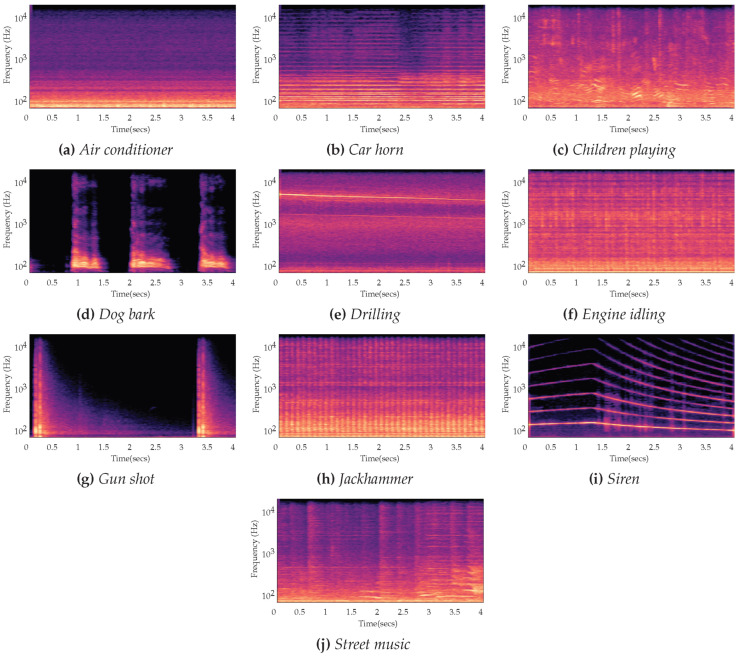
The behaviours of time-dependant frequencies captured in spectrogram representation from different classification schema. Reprinted from ref. [97].

The wavelet components from acoustic data, temperature changes and thermal location from DTS data, low-frequency acoustic signal, spectrogram plot, F-K plot, as well as the mean and variance from a time window of DAS and DTS data, have been used as the main features for modelling distributed fibre optic data with machine learning. The spectrogram plot from acoustic data [97], for example, can provide rich interpretations of different classification schema, depicted in Figure 13. F-K plots, on the other hand, are the representation of the SoS values, which have a strong correlation with the phase-fraction information of multiphase fluid. Providing a clear V-shape sign from F-K plots, however, is not a trivial process. It requires a longer range of spatial channels, as well as sufficient acoustic fidelity within each channel [48].

### 5.3. Learning Algorithms

Machine learning algorithms are used to recognize pattern of an object given the input features (handcrafted or otherwise). Support Vector Machine [57,59], Kalman Filter families (including the Extended Kalman Filter (EKF) and Ensemble Kalman Filter (EnKF) [41,107]), and Neural Network families [57,106,108], are some of the machine learning algorithms that can be used for multiphase flow characterisation.

The SVMs use kernel functions to transform the input features to the higher dimension, therefore, the data can be linearly separated in the new dimension. The SVM kernel functions work as the non-linear feature transformation to allow the SVM algorithm to handle non-linear systems that are often the case in the petroleum industry [42]. The EKF and EnKF, on the other hand, work by tracking the dynamic model of the data using the state-space estimation based on variance-covariance matrices in time. The original Kalman Filter was developed for the linear system, while these extension algorithms avoid the linearization by estimating the covariance matrix instead of using the true matrix, called ensembles. Both EKF and EnKF are used due to their robustness to noise and data corruption, fast implementation, and their dynamic non-linear estimation can provide accurate results. The study by Loh et al. [41] shows that the EnKF updated model can provide a more accurate prediction compared to the ones without EnKF, allowing possible application for a realtime monitoring solution.

**Figure 14:**
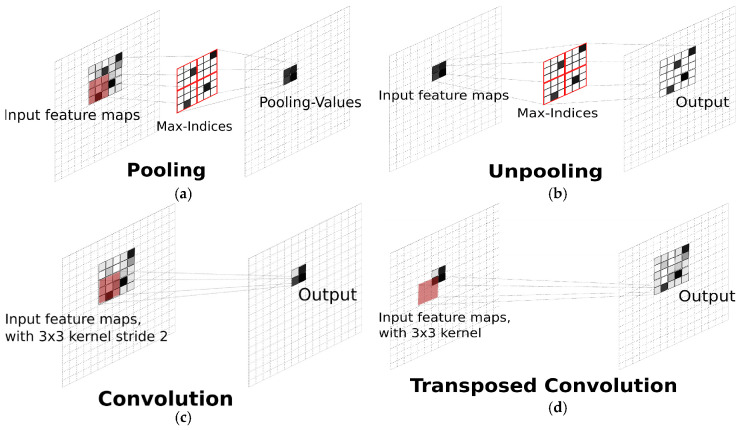
Illustration of (**a**) pooling, (**b**) unpooling, (**c**) convolution, and (**d**) transposed convolution operations in NN models. Reprinted from ref. [109].

Lastly, the NN families use a stack of weighted linear structures (called layers) with intermediate non-linear functions to perform automatic classification/prediction. There are several operations that can be used in the NN-models, including but not limited to (a) pooling, (b) unpooling, (c) convolution, and (d) transposed convolution operation, depicted in Figure 14. The NN structures consist of thousands of parameters that are optimized by using the gradient descent algorithm and backpropagation parameter update operation. ANN, CNN, and RNN are some types of NN algorithms that can be used for multiphase estimation. For example, Vahabi et al. [37] used CNN to perform phase classification using the F-K plot from the DAS data as input, providing a high accuracy classification (99.3% accuracy on test data). It should be noted that the CNN can model different type of data representation, e.g., F-K and spectrogram plots, depicted in Figure 6 and Figure 11, respectively.

It is worth mentioning that the NN families and SVM algorithms are considered black-box approaches. It means that a model generated by these algorithms is hard to interpret, and often the results give limited insight into the underlying physical processes. Therefore, several works [42,60] have tried to combine the physical features and first-principle methods with the black-box algorithms to ensure the reliability of the predictions, and facilitate the building of trust among stakeholders within the industry.

A summary of machine learning algorithms that have been used with distributed fibre optic data for flow rate and multiphase estimation is provided in Table 2. Silkina [36] used ANN to correctly identify the flow conditions of multiphase fluids, providing almost 100% accuracy. A simple two-layer MLP was used to classify 11 different classes combining water and pine oil with different air-flow rates. However, the random split between training and test data on a sequentially generated dataset indicates a potential information leakage, undermining the overall accuracy performances. Park et al. [110] considered the total spectral power of the signal within a bounded range from DAS as a regression model along with the measured flow rates. The model was trained using a robust regression algorithm to reduce the effect of corrupted data and outliers. Even though the presented results are limited in term of accuracies and performances, they addressed important issues within modelling corrupted data and addressing outliers within the acoustic data.

Ghahfarokhi et al. [104] used an averaged daily data from 1320 DTS measurements along the lateral of the gas-producing well in the Marcellus Shale, in Northern West Virginia to forecast daily gas production. An MLP model was trained and deployed, and Sensitivity Analysis (SA) was conducted to analyse weight behaviour. Similar to [104], Bhattacharya et al. [112] used DAS and DTS among other datasets to predict daily gas production using ANN, SVM, and RF. A high accuracy (96%) was achieved by employing 18 features to RF model for prediction. However, the utilization of DAS and DTS in the project was limited, since they were presented as spatially averaged point measurements before being fed into to the main classifier. Therefore, the results did not fully benefit from the the distributed and real-time nature of the sensors.

Another interesting work was presented by Vahabi et al. [37,40]. The DAS data, collected from real oil, water and gas well pipes under the sea, was used to identify fluid types [37] and to estimate fluid flow velocities [40] using machine learning algorithms. The F-K transformation technique was employed to provide input data for the CNN and ANN models to classify the type of fluid in pipes. The highest accuracy of 99.3% can be achieved by CNN, which indicates a potential for further classifying multiphase fluids using DAS data under a real production environment. Other machine learning algorithms, such as Cross-correlation, K-Means, and Radial Integration, were also employed to determine fluid flow velocity in pipes. Flow velocity from the wellhead was used as the true label, and with some physical assumptions, the machine learning algorithms performed quite well on estimating the flow velocity from input data derived according to the F-K transformation [40].

### 5.4. Inference and Uncertainty Estimation

The inference process generates predictions as the final outcome and (often) is coupled with prediction uncertainty estimation. For a realtime solution, inferring results from a machine learning model not only requires the model to generate accurate prediction, but also to deliver the results in timely manner. The CPU implementation of ANN model can perform automatic flow regime classifications within 0.02 s, while the CNN model can provide them with 99.3% accuracy within 0.01 s [37]. It must be mentioned that for a realtime monitoring solution, the time for data acquisition and preprocessing must be included in the overall prediction times. And those, however, are still a bottleneck for the CNN implementation, as mentioned in [37].

In addition to the inference time, the uncertainty estimation is also an important factor for delivering monitoring solutions based on the machine learning algorithm. This is partly due to the stochastic nature of machine models when making the prediction and the black-box property of some machine learning algorithms. Therefore, having the prediction coupled with uncertainty estimation can increase the confidence and reliability of the prediction from a machine learning model.

Several techniques can be used to measure the prediction uncertainties. These techniques include Bootstrap algorithm, Bayesian statistic, and Dropout technique for NN-based models [113]. These techniques use input data, posterior information, parameter values, and the like, as the control variables to measure the changes in the final predictions. The more varied the predictions given the changes in the control variables, the larger its prediction uncertainty. On the other hand, the more uniform the predictions given those changes, the smaller the prediction uncertainty of the aforementioned model.

## 6. Discussion and Comparison

Table 1 compares several techniques that have been used for modelling distributed fibre optic data for production monitoring in the petroleum industry. It is important to highlight that most of these methods were tested on confidential datasets, from real production fields and flow-loop experiments. Some of them can provide high accuracy predictions on their predefined objectives, either for flow rate estimation or single/multiphase classification. It should be noted that the Doppler Effect technique has been used extensively as the state-of-the-art method for flow rate estimation, while the ANN-based machine learning algorithms have been used for flow regime and multiphase classification.

In this section, we will discuss how some of these methods perform for realtime monitoring in the real well environments, as well as providing future research directions for multiphase flow estimation. We will first summarize the main differences between physical flow modelling and machine learning algorithms. We then discuss the performance and limitations of these methods, and finally lay out potential future research directions for data-driven machine learning algorithms on distributed fibre optic sensors.

### 6.1. Physical Flow Modelling and Machine Learning Algorithms

One of the main differences between physical flow models and machine learning approaches is within the learning process. The physical models use the hydrocarbon mixture database to estimate the multiphase flow, while the machine learning techniques use the so-called learning algorithm to extract patterns from the data. It is important to note that the hydrocarbon mixture database is a robust, accurate, and well-proven reference data that can provide high accuracy multiphase information. In the real field operations, especially in the HPHT environments, relying solely on this database limits the ability of the physical flow modelling algorithm to provide reliable distributed monitoring solution. This is due to the limitations in the modelling process itself, that must often make assumptions concerning system complexity such ignoring the influence of irregular surfaces inside the pipes, the nature of surrounding HPHT environments, and complex fluid behaviours such as the formation of hydrate and emulsion phases.

Data-driven machine learning algorithms, on the other hand, work by analyzing patterns in the data and can model (theoretically) any complex system accurately, even with limited to none information of the underlying system (see universal function approximations [114]). Therefore, those techniques are capable to approximate and model any well-understood phenomenons, as well as address the unknowns. This capability is advantageous for fluid flow estimation, especially in the field of distributed fibre optic sensors, where the data and their references/labels are abundant. For example, with an adequate number of layers and enough data points, the NN-based algorithms can provide a very accurate prediction with high certainty. In some cases [115], they can perform better then human predictions. It is known that training a large structure of NN model with large volume of data can be extremely time consuming and resource intensive [116], not to mention the limitation of those methods to provide clear explanation of their results.

Due to their black-box nature, the NN-algorithms mentioned in Table 2 mostly ignore the temporal correlations among data points. It has been known that temporal correlation is an important characteristic for modelling sequential and time-series data. Several state-of-the-art algorithms for sequence modelling, including Convolutional LSTM [117] and Attention model [118], have provided significant improvement in terms of accuracy, by considering the temporal dependencies within the input data. In addition, a graphical model, such as Conditional Random Field (CRF) principle [119], can also be used to cross-correlate the spatiotemporal structure of the distributed sensors, tying together the spatiotemporal relationship among each spatial channel and their neighbouring channels in the spatial domain as well as in the time domain.

Another contrast between physical flow models and machine learning algorithms is the way features are generated. The physical models use physical formulations and first principle methods to generate representative features, while the majority of the machine learning algorithms (in Table 2) use NN-based feature generators. Physical formulations are fast and reliable methods for understanding and explaining the fluid dynamics, hydrocarbon mixture phenomenons, and thermophysical events. NN-based feature generators, on the other hand, have the ability to learn from the data without being boxed by rigid-known formulations. They can learn and address the unknowns and (eventually) provide robust and accurate estimations [120].

### 6.2. Challenges

As can be seen in Table 2, there has been limited research focusing on estimating multiphase flow by combining distributed fibre optic sensor and machine learning technologies. This is in part, due to the complexity of the physical system but perhaps more significantly, a lack of access to relevant annotated distributed fibre optic datasets. The NN-based machine learning algorithms are data intensive techniques that work well when sufficient data are available for training. Unfortunately, to the best of our knowledge, only limited amount of available DAS or DTS datasets that can be used for developing multiphase fluid flow characterisation techniques. Thus, collaborations with oil and gas companies as well as research institutions are a necessity for advancing this field further. For many applications, easy access to annotated datasets is a trend that is helping to accelerate machine learning research, for example, ImageNet for image classification [121], Pascal VOC2012 for image segmentation [122], ISPRS Vaihingen dataset for remote sensing [123], and KITTI dataset for autonomous vehicle applications [124], have become the backbone of many advances within those research areas.

Another challenge for multiphase flow characterisation using distributed fibre optic sensors is providing fast and near real-time classification. This is mostly due to the large volume of DAS/DTS data that are being generated and used for processing. As was mentioned in [37], 40 TB of DAS data were generated during 24 h of measurements, this is an equivalent to around 28 GB of data per minute that must be processed to provide a real-time monitoring capability. Resource intensive data processing algorithms and high performance infrastructures are required in order to model such large data within the machine learning environment.

In addition to the dataset access problem and real-time processing issue, the black-box nature of the NN-based algorithms also limits the progress and adaptation of the machine learning techniques for multiphase fluid flow characterisation. Reservoir engineers and stakeholders within the field have some reservations with the use of black-box algorithms for estimating the multiphase fluid flow compared to the first principle-based methods and physical flow modelling techniques [42].

### 6.3. Relevant Work from Other Industries

Estimating the phase fraction and flow velocity using acoustic sensors, Doppler Effect, and machine learning is not only applicable for multiphase flow estimation within the oil and gas industry. The medical industry, for example, has used similar technology to detect anomalies and blood flow rate inside the veins using techniques called Biomedical Photoacoustic Imaging (BPI) [125] and Venous Doppler Ultrasound (VDU) [126]. The BPI, for example, uses sound wave formation and optical absorption in biological tissues to form a biomedical image modality that can be used to measure hemoglobin concentration and oxygen saturation. Combining BPI and machine learning have also been explored, for example, to remove the photoacoustic reflection artefacts [127], to measure the prediction uncertainty [128], or to reconstruct the photoacoustic faster using ANN [129].

The transportation industry, on the other hand, has also been using DAS and machine learning for analysing traffic flow and detecting objects within the flow [31,130]. The phase-fluid components and fluid flow rates can be thought as analogues to traffic object movements and traffic flow respectively. Blood property concentration and blood flow rate are similar analogues from the medical industry. By mimicking the process and reconstructing the technologies and advances from other industries, we can further accelerate the research for multiphase fluid flow estimation within the oil and gas and process industries. Table 3 shows several machine learning algorithms that are used for modelling distributed sensor data from different fields and industries.

### 6.4. Future Research Directions

Despite the existence of distributed temperature measurements and thermal models for more than 20 years, as pointed out in [34], DTS is limited in terms of characterizing three-phase fluids. DAS on the other hand, with the ability to measure rapidly varying dynamic physical properties, offers a richer variety of possibilities. As a result, the current state-of-the-art for distributed fibre optic multiphase estimators is becoming increasingly weighted towards DAS, and this is trend is likely to continue into the future.

Table 2 and Table 3 encompass the rapid development of modern NN-based algorithms on modelling distributed fibre optic data. Getting access to data relevant for applying such techniques to multiphase fluid flow characterisation is key to accelerating this field where there is clear potential for significant advances. Such advances could be effectively done through interdisciplinary collaborations between industry and academia, with natural mechanisms for monitoring the progress and benchmarking the quality, accuracy, and processing time of new data modelling techniques.

In order to provide high accuracy multiphase fluid flow characterisation, modelling the spatiotemporal aspects of distributed systems should also be considered as an interesting research direction. The fibre optic sensor as a monitoring system is a spatiotemporal structure consisting of large volume of temporal data with dense spatial resolution. The Attention Model with its positional encoder combined with the RNN architecture for its temporal encoder could potentially help understand those spatiotemporal structure resulting on a higher accuracy classifier.

The machine learning algorithms, especially the NN-based algorithms, have the ability to extract useful features from vast amounts of data while providing high accuracy predictions, and thus can simplify the data modelling process. Despite their potential effectiveness for certain applications, their black-box nature can present challenges with regard to gaining stake-holder trust and confidence. This challenge is less for more conventional approaches relying on physical flow models that are based on scientifically proven phenomena through experiment. The combination between the two will greatly advance this field on providing high accuracy prediction with explainable outcome, for example, (1) using the physical flow components as input features for the machine learning algorithm, (2) using the physical flow models for self-calibrating machine learning predictions, or (3) using the machine learning algorithms to validate the first principle methods.

## 7. Summary

Accurate and real-time multiphase fluid flow characterisation techniques employing distributed measurement capabilities will provide a game-changing functionality for production optimization in the oil and gas industry. The state-of-the-art in terms of physical flow modelling techniques and machine learning algorithms has been presented and discussed in this paper. An extensive review and comparative summary of the structure of the state-of-the-art has been provided. The characteristics, performance, and trade-offs between different algorithms were discussed. A comprehensive analysis of the potential of machine learning algorithms for modelling the fibre optic sensor data for multiphase estimation has been included. Finally, potential future research directions for multiphase fluid characterisation using distributed fibre optic sensors and machine learning algorithms were discussed.

## Figures and Tables

**Table 1:** Comparison of physical flow modelling techniques used with distributed fibre optic sensors for flow rate estimation and multiphase classification.

Modelling Technique	Year	Data Sources	Note
Doppler effect [85]	2012	Field surveys with DAS	Early work on DAS for well and reservoir monitoring.
Doppler effect and Root Mean Square (RMS) of acoustic energy [48]	2014	Field trial with DAS	Early implementation of DAS on real oil field.
SoS and eddy velocity estimation [86]	2015	Flow-loop experiment with DAS	Ability to visualize the generation and convection of eddies using waterfall plot of distance versus time.
SoS and J-T coefficient value matching [8,34]	2016	Production oilfield with DAS and synthetic DTS	Integral image algorithm for estimating SoS of multiphase fluids and Ability to accurately measure two-phase flows.
Forward model [87]	2017	Simulated DAS	Simulating DAS data taking into account formation wellbore properties, flow characteristic, noise processes and optical fibre parameters.
Thermal-and-hydraullic modelling [88]	2018	DAS and DTS	Thorough analysis on combining DAS and DTS data for identifying gas flow.
Multiphysics analysis and clustering optimization [89]	2019	Flow-loop experiment	Applied on steam flow profiling experiment with high resolution DTS and DAS data.
Statistical analysis and SAGD modelling [90]	2019	Flow-loop experiment and simulation model	Designing and commissioning an advanced multi-phase flow injection experiment.
SoS analysis [91]	2019	DAS and DTS	Applicable for HPHT horizontal gas producer.

**Table 2:** Comparison of machine learning algorithms on using distributed fibre optic sensors for flow rate estimation and multiphase classification.

Modelling Technique	Year	Data Sources	Note
ANN [36]	2014	Flow loop experiment with DAS	Early report and experiment for using DAS data and ANN for flow regime classification and flow rate estimation.
ANN [87]	2017	Simulated DAS	The wavelet coefficients are the input and flow pattern are the output.
Robust regression and band switching algorithm [110]	2018	DAS	Frequency Band Extracted (FBE) bands analysis is used to improve the prediction accuracy.
MLP [104]	2018	Gas producing well with DAS and DTS	Mainly focus on using DTS for forecasting gas production while DAS data was only recorded during hydraulic fracturing of the well.
Decision Tree, Adaptive Boosting, and Random Forest (RF) [111]	2019	Real field DAS	Training was conducted under limited amount of data.
ANN, SVM, and RF [112]	2019	Gas production well with DAS and DTS	A well defined data-driven machine learning experiment, including the use of sensitivity analysis for analyzing feature importance.
ANN [96]	2019	DAS	Autoencoder ANN is used for modelling acoustic and flow rate data.
CNN, ANN [37]	2019	Real well underwater DAS	Resulting on high accuracy flow regime classification from F-K images of DAS data.
Cross-correlation, K-means, and Radial integration [40]	2020	Real well underwater DAS	Providing fast flow velocity estimation from a large volume of DAS data.

**Table 3:** Recent work concerning machine learning algorithms for modelling distributed fibre optic sensor data for non-multiphase fluid flow objectives.

Algorithms	Year	Objectives	Note
Gaussian Mixture Model (GMM) and Hidden Markov Model (HMM) [131]	2019	Pipeline integrity threat detection	The contextual information at the feature level was incorporated in a Gaussian Mixture Model and Hidden Markov Model (GMM-HMM)-based pattern classification system for acoustic trace decision strategy.
*k*-Nearest Neighbor (*k*NN) and SVM [132]	2019	Event identification	The disturbance events, such as knocking, pressing, watering, climbing, and false disturbance event, are identified for 25.05 km long OTDR system using combination of *k*NN and SVM.
HMM [133]	2019	Pipeline safety monitoring	The HMMs were trained to identify sequential state process of events and extract the temporal information of the data, and provided an average accuracy of 98.2%.
Dual Path Network [134]	2019	Railway safety monitoring	The proposal provides proof-of-concept on using distributed sensor and machine learning algorithm for actual railway safety monitoring. The F1-scores for all classes reached up to 97% in the test data.
CNN [135]	2019	Microseismic event detection	The synthetic microseismic events injected into recorded ambient noise and was trained using CNN to detect seismic events in the test DAS data.
NN [29]	2019	Fracture-hit detection	The NN was trained on Low-frequency distributed acoustic sensing (LFDAS) to detect fracture hits to monitor wells during hydraulic fracturing operations.
DNN [136]	2019	Human movement identification	The DAS signal was enhanced using ultrafast laser; the data was trained using supervised and unsupervised machine learning algorithms to detect human movement and pipeline monitoring.
SVM [137]	2020	Train tracking	The vibrations of moving objects are used to identify and track trains in real-time; the algorithm runs on GPU to speed up the calculations.
CNN, LSTM, K-means [138]	2020	Human locomotion identification	High spatial resolution and bandwidth data was shown to be effective on increasing the machine learning accuracy.
LSTM [139]	2020	Railway intrusion detection	A real field experiment with noise background sound was conducted in this study, resulting on shortening the average detection response time to 8.25 s.
Random Matrix Theory (RMT) [140]	2020	Event activity detection	Events were detected along with their location on the fibre, then they were extracted from the random noise using Spiked RMT models.
CNN [141]	2020	Earthquake detection	The CNN shows a promising results for providing a reliable earthquake detection despite low signal-to-noise ratio of the fibre telecom infrastructure.

## Data Availability

Not applicable.

## Abbreviations

The following abbreviations are used in this manuscript:

ANN	Artificial Neural Network
BPI	Biomedical Photoacoustic Imaging
CNN	Convolutional Neural Network
CRF	Conditional Random Field
DAS	Distributed Acoustic Sensor
DS	Distributed Sensor
DTS	Distributed Temperature Sensor
EIT	Electrical Impedance Tomography
EKF	Extended Kalman Filter
EnKF	Ensemble Kalman Filter
F-K	Frequency and Wavenumber
FBE	Frequency Band Extracted
FFT	Fast Fourier Transform
GAN	Generative Adversarial Network
GMM	Gaussian Mixture Model
GPU	Graphical Processing Unit
GVF	Gas Volume Fraction
HMM	Hidden Mixture Model
HPHT	High Pressure High Temperature
ICD	Inflow Control Device
ICV	Inflow Control Valve
ISPRS	International Society for Photogrammetry and Remote Sensing
IU	Interrogation Unit
J-T	Joule Thomson
KF	Kalman Filter
KITTI	Karlsruhe Institute of Technology and Toyota Technological Institute
kNN	k-Nearest Neighbor
LFDAS	Low-Frequency Distributed Acoustic Sensor
LSTM	Long Short-Term Memory
MLP	Multi Layer Perceptron
MPFM	Multiphase Flow Meter
MSEEL	Marcellus Shale Energy and Environment Laboratory
NCS	Norwegian Continental Shelf
NIST	National Institute of Standards and Technology
NN	Neural Network
OTDR	Optical Time-Domain Reflectometer
PC	Personal Computer
PCA	Principle Component Analysis
RF	Random Forest
RMT	Random Matrix Theory
RNN	Recurrent Neural Network
SA	Sensitivity Analysis
SADG	Steam Assisted Gravity Drainage
SoS	Speed of Sound
SNR	Signal to Noise Ratio
SVM	Support Vector Machine
VDU	Venous Doppler Ultrasound
VFM	Virtual Flow Meter
VOC2012	Visual Object Classes Challenge 2012
VSP	Vertical Seismic Profiling
WLR	Water in Liquid Ratio
